# Bead-Selected Antitumor Genetic Cell Vaccines

**DOI:** 10.4137/cmo.s586

**Published:** 2008-03-25

**Authors:** MJ Herrero, Botella R, Algás R, FM Marco, SF Aliño

**Affiliations:** 1 Gene Therapy Group, Dpto. Farmacologia, Fac. Medicina, Univ. Valencia, Valencia, España; 2 Servicio Dermatologia, Instituto Valenciano Oncologia, Valencia, España; 3 Servicio Radioterapia, Hospital Clínico Universitario, Valencia, España; 4 ASAC Pharmaceutical International, Alicante, España

**Keywords:** cancer vaccines, gene therapy, bead selection, GM-CSF, B7.2

## Abstract

Cancer vaccines have always been in the scope of gene therapy research. One of the most successful approaches has been working with genetically modified tumor cells. However, to become a clinical reality, tumor cells must suffer a long and risky process from the extraction from the patient to the reimplantation as a vaccine. In this work, we explain our group’s approach to reduce the cell number required to achieve an immune response against a melanoma murine model, employing bead-selected B16 tumor cells expressing GM-CSF and B7.2.

## Introduction

Genetically modified tumor cells are a very interesting approach with high potential in the field of cancer vaccines (Dunussi-Joannopoulos et al. 1998; Kim et al. 2000; Mastrangelo and Lattime, 2002). In order to fight any type of cancer, the most reasonable strategy seems to be the use of the same tumor cells that we want to eliminate. Indeed, the transfection of these cells with immune-stimulating molecules as the cytokine GM-CSF (granulocyte and macrophage colony stimulating factor) has also been employed (Dunussi-Joannopoulos et al. 1998; Jaffee et al. 1998; Shi et al. 1999; Borrello and Pardoll, 2002; Moret-Tatay et al. 2003; Serafini et al. 2004; Herrero et al. 2006; Moret-Tatay et al. 2006). However the signalling pathways of the immune system are very complex and in the last few years many studies have tested different combinations of cytokines with other molecules in order to improve antigen presentation, as for instance the membrane surface costimulatory molecule B7.2 (Chong et al. 1998; Kim, 2001; Mukherjee et al. 2001; Parney et al. 2002; Zajac et al. 2003; Pizzoferrato, 2004).

Working with autologous tumor cells to prepare an antitumor vaccine is quite a difficult task, since usually there are several critical stages to overcome: extracting cells from the patient, genetically modifying the cells, keep them in culture safely and expand them to the desired amount to ensure sufficient antigen and transgene for the treatment to work. For all these reasons, minimizing the quantity of tumor cells required in cancer vaccines is a great challenge (Farray and Clark, 2006; Herrero et al. 2006; Copier et al. 2007; Herrero et al. 2008).

In this technical note we describe the tumor cell vaccine approach of our group employing magnetic bead selection of nonviral transfected tumor cells. We have previously achieved total survival of B16 melanoma-bearing mice in a preventive vaccine model using freshly GM-CSF transfected (non-selected) B16 cells (Moret-Tatay et al. 2003; Herrero et al. 2006; Herrero et al. 2008). Now we wanted to take advantage of the fact that membrane surface B7.2 molecule has also already demonstrated its usefulness in some antitumor vaccine models. We therefore transfected B16 cells with a bicistronic plasmid containing both *m-gmcsf* and *m-B7.2* genes. Thus, we expect that the truly transfected cells, expressing B7.2 on their surface, would also express GM-CSF. This would allow bead selection of the B7.2 transfected cells from the heterogeneous population of transfected and non-transfected cells following a usual transfection procedure. With this new approach, we could study the possibility of reducing the number of cells required for a successful antitumor vaccine.

## Materials and Methods

### Plasmids

The p2F m-gmcsf+m-B7.2 plasmid was derived from the pVITRO2 base plasmid (Invivogen, Toulouse, France), with the *m-gmcsf* plus *m-B7.2* genes. pVITRO2 allows the coexpression of two genes and contains two human ferritin composite promoters, FerH (heavy chain) and FerL (light chain), combined to the SV40 and CMV enhancers respectively, and the resistance gene to hygromycin.

All plasmids were amplified in *Escherichia coli* DH5α, in selective LB broth (Pronadisa, Madrid, Spain) and extracted with the Qiagen Giga Endo-free kit (Izasa SA, Barcelona, Spain), quantified by spectrophotometry and tested by electrophoresis to confirm their integrity and purity.

### Cells and transfection procedure

B16 murine melanoma cells have been used in all of the experiments. These cells are syngeneic with the animals used for vaccination, i.e. C57BL/6 mice (Harlan, Gannat, France).

B16 cells are adherent cells that are grown in flasks with DMEM (Dulbecco’s modified Eagle’s medium) (Sigma, Madrid, Spain), supplemented with 10% heat inactivated fetal bovine serum (FBS) (Biomedia, Boussens, France), penicillin (100 U/ml) and streptomycin (100 μg/ml). The cells are cultured in a humidified incubator with 5% CO_2_ at 37 ºC, and are detached from the flasks with Trypsin-EDTA.

Lewis lung carcinoma, 3LL, cells were also employed, cultured under the same conditions as described.

The B16 cells employed for the vaccines were transfected by means of a chemical procedure based on PEI 25 KDa (polyethyleneimine. Sigma, Madrid, Spain) polyplexes (DNA:PEI, 1:1.41) with 20 μg/ml p2F plasmids, as previously described (Moret-Tatay et al. 2003; Guillem and Aliño, 2004; Herrero et al. 2006; Moret-Tatay et al. 2006). The transfection percentage with this method lies between 20%–40% of total cells (data not shown), as observed using the reporter EGFP gene. Cells were transfected when more than 80% confluence was reached in their flasks. Tumor cells were irradiated 72 hours post-transfection with 150 Gy, and then frozen in DMSO 5% in FBS and kept at −80 or −150 ºC until use.

3LL cells employed as vaccine were irradiated with 50 Gy dose.

### ELISA of m-GMCSF

GM-CSF production of the transfected B16 cells is determined by Enzyme Linked Immunosorbent Assay (ELISA), performed on supernatant samples of the culture media taken 72 hours post-transfection and prior to cell detachment and irradiation, having changed the media every 24 hours. The BD OptEIA ELISA kit for m-GMCSF (Pharmingen, BD Biosciences, Madrid, Spain) was used. The time-point of 72 hours was chosen on the basis of prior experimental results, assessed to study cytokine production over time, and using the referred transfection conditions (Moret-Tatay et al. 2003; Guillem and Aliño, 2004; Herrero et al. 2006; Moret-Tatay et al. 2006), in order to achieve adequate production according to the literature (Borrello and Pardoll, 2002, Serafini et al. 2004).

### Cytometry of m-B7.2 expression

Flow cytometry was performed to confirm the presence of m-B7.2 on the surface of the transfected cells. At 72 h post-transfection, cells were harvested and pelleted in aliquots of 500,000 cells, washed and incubated in ice for at least 30 min. in 200 μl PBS-FBS (2.5%) -azide (0.01%) solution of primary antibody (1 μg/million cells of biotin-conjugated rat anti-mouse CD86 monoclonal antibody (Pharmingen, BD, Madrid, Spain). Then, cells were washed twice in 1 ml PBS-azide and incubated in ice and darkness with the secondary antibody (0.5 μg/million cells): Streptavidin-Fluorescein Isothiocyanate Conjugate (FITC) (Pharmingen, BD, Madrid, Spain).

Finally, cells were washed twice and resuspended in 500 μl PBS to be passed through the cytometer (Coulter Epics, XL; Beckman Coulter, Madrid, Spain) in order to analyze fluorescence in the populations, to discriminate the truly transfected cells. To avoid considering autofluorescence or nonspecific binding as positive results, several controls were tested. The groups were: a) non-transfected wild type B16 cells, without any antibody; b) non-transfected wild type B16 cells incubated with both antibodies; c) B16-p2F m-gmcsf + m-B7.2 transfected cells, without any antibody; d) B16-p2F m-gmcsf + m-B7.2 trans-fected cells, incubated only with primary antibody; e) B16-p2F m-gmcsf + m-B7.2 transfected cells, incubated only with secondary antibody; and finally f) B16-p2F m-gmcsf + m-B7.2 transfected cells, incubated with both primary and secondary antibody.

### Bead selection and purification of transfected cells

This process is carried out by means of the Automacs device for magnetic cell separation (Miltenyi Biotec, Madrid, Spain), according to the manufacturer’s instructions and employing the Automacs columns. At 72 h post-transfection, cells are harvested and the Streptavidin Microbeads protocol is then followed (Miltenyi Biotec, Madrid, Spain). Briefly, cells are incubated with the primary biotinylated antibody, as described for flow cytometry. Then, cells are incubated with streptavidin magnetic beads, as secondary antibody and finally they are passed through the Automacs column, using “Posseld 2” program, which gives us two eluted fractions, one containing the transfected cells, expressing B7.2 on their surface and thus also GM-CSF, and the other containing the non-transfected cells. Lastly, positive cells are counted and frozen until the time of the vaccination experiments.

### Vaccination procedure

C57BL/6 mice (8–10 weeks old) kept under standard laboratory conditions were housed 5 mice per cage. All the experiments were approved by the Biological Research Committee of the University of Valencia (Valencia, Spain). In all cases, mice were vaccinated (right leg) with a single dose per week, in weeks −3, −1 and +1 (days −21, −7 and +7), with respect to tumor injection (day 0) with 10^5^ wild type B16 cells in the left leg or 10^5^ wild type 3LL cells, where indicated.

The number of cells employed in each vaccine dose represented only the truly transfected cells of previous experiments with non-selected cells, where total survival of the treated animals was achieved (Moret-Tatay et al. 2003; Herrero et al. 2006; Herrero et al. 2008). Thus, we had to vaccine only with 20% of the previous doses (this being our expected percentage of truly transfected cells in that case). This meant vaccinating with 40.000 selected cells per mouse, per dose, in 100 μl DMEM. We also vaccinated with the double dose, 80.000 selected cells, and since this was still a reduced number of cells, we took the opportunity of exploring how these cells would work in a model where another type of tumor cells constituted the real target and B16 were just the bystander cytokine-producing cells. To this effect, we vaccinated with 80.000 B16 selected cells + 320.000 3LL cells.

In all vaccination experiments, blood samples were taken from all the animals, pooling blood from the animals from the same group at each time-point. The samples were taken on days −22 (before any manipulation of the animals, serving as base level or control in each group), −1 (the day before tumor implantation), and +15 (one week after the third and last dose was administered). Plasma was obtained by centrifugation at 3000 rpm for 5 min. and kept at −20 ºC until use.

### Tumor growth measurement and survival

Tumor growth in mice was monitored visually and measured with a caliper in two dimensions: A, the long diameter and B, the short diameter. Tumor volume was calculated with the formula V = (A × B^2^)/2, and expressed in mm^3^. Animals were collected at their death date to construct the survival curves.

### Specific anti-TMP IgG ELISA

Measurement of IgG and IgG1 and IgG2a subclass antibodies to TMP (Tumor Membrane Proteins) was performed in serum samples by specific ELISA, as previously described (Herrero et al. 2006; Herrero et al. 2008). TMP is an extract of the hydrophilic membrane proteins of the irradiated B16 cells; thus, so with this ELISA, we test the specific response to our vaccine treatment, discarding any other non-specific immune responses (Bordier, 1981; Herrero et al. 2006; Herrero et al. 2008).

Plates were coated by overnight incubation of TMP at 0.8 μg/ml in carbonate buffer, pH 9.6. The next day, plates were neutralized with 1% BSA solution before addition of serum samples. For analysis, sera were diluted in dilution buffer (PBS-BSA 1%-Tween 20 0.1%) at 1/1000 for total IgG and IgG1 subclass and at 1/100 for IgG2a. Bound antibodies were detected with goat antisera to total IgG (Biocheck, Foster City, USA) at 1/10000 or mouse IgG subclasses at 1/1000 (Sigma, Mouse monoclonal isotyping reagents, Madrid, Spain), followed by 1/5000 dilution of biotinylated rabbit antiserum to goat IgG (Sigma, Madrid, Spain) and streptavidin coupled to horseradish peroxidase (Sigma, Madrid, Spain). Plates were developed with a mixture of orthophenylenediamine (OPD, Sigma, Madrid, Spain) and hydrogen peroxide (Fluka-Sigma, Madrid, Spain), and read at 492 nm. All samples were assayed in duplicate, allowing estimation of mean OD value and standard deviation.

## Statistical analysis

To statistically compare the results of tumor growth inhibition in the different treatment groups, a two-way ANOVA was employed, with Bonferroni post hoc testing (95% confidence interval, 95%CI), expressing statistically significant differences on the basis of P < 0.05, P < 0.01 and P < 0.001. The same test was applied to the results of ELISA assays.

For survival significance, we used the Kaplan-Meier survival curves and logrank non-parametric test.

All the tests and graphs were performed with Graph Pad Prism 4 software®.

## Results

### Determination of m-B7.2 production

**Figure 1:**
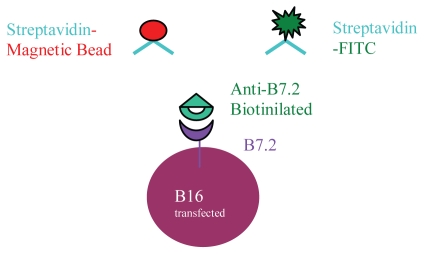
Binding strategies to B7.2 B16 cells transfected with the plasmid p2F m-gmcsf+m-B7.2, will secrete GM-CSF into the medium and will also express B7.2 protein on their surface. The biotinylated anti-B7.2 antibody allows not only binding of the cells to streptavidin-FITC to visualize them in cytometry assays, but also binding to streptavidin-magnetic beads, what let us separate these cells from the non-transfected ones.

The strategies for the detection of B7.2 expression are illustrated in Figure 1. We employed a biotinylated anti-B7.2 antibody as the first step for two kinds of experiments: first, to check the transfection efficacy by flow cytometry, using streptavidin-FITC as second step; second, to separate the positive (transfected) cells by magnetic beads selection, using streptavidin beads.

**Figure 2:**
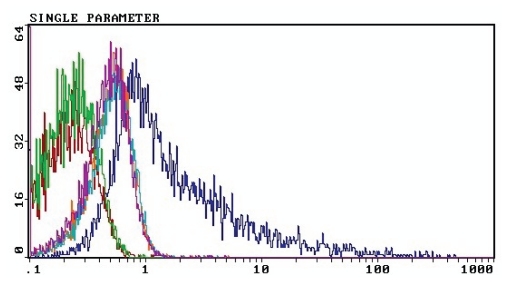
Flow cytometry of B16 cells transfected with p2F m-gmcsf+mB7.2 The following B16 cell groups were passed through the cytometer and their graphs overlayed in a single one: a) B16 wild type cells, non-transfected and with no reagents incubation; and b) B16 wild type cells, non-transfected and with both the primary antibody and streptavidin-FITC. These two groups form the left peak. The central peak is formed by c) B16 transfected cells with p2F m-gmcsf + m-B7.2, without the reagents incubation, d) B16 transfected cells with p2F m-gmcsf + m-B7.2, incubated only with the primary antibody and e) B16 transfected cells with p2F m-gmcsf + m-B7.2, only with streptavidin-FITC. Finally, f) B16 transfected cells with p2F m-gmcsf + m-B7.2, incubated with both primary antibody and streptavidin-FITC, corresponds to the right peak. The graph represents the green fluorescence in X versus count number in Y.

The efficiency of m-B7.2 expression on B16 cell surface, 72 h after transfection, was evaluated by flow cytometry. The results are summarized in Figure 2, which plots counts (Y) versus fluorescence (X), and where we can identify specific surface expression of m-B7.2 in the right peak of the figure, whereas the middle and left peaks correspond to the transfected and non-transfected control cells, respectively.

The right area of the right peak, beyond the intersection with the middle peak, represents the proportion of transfected cells that are also fluorescent, i.e. the cells that express m-B7.2 on their surface. This area is approximately 40%–50% of the right peak, which was our expected transfection efficacy, according to our previous experiments (data not shown).

### Purification of transfected cells by means of magnetic beads

As explained above, 72 h after transfection with p2F m-gmcsf + m-B7.2, the cells were incubated with biotinylated anti-B7.2 and later with streptavidin beads, according to the manufacturer’s protocol. The selected cells also produced GM-CSF, as tested by ELISA, approximately ranging from 90 to 175 ng/10^6^ cells.

The efficiency of the transfected B16 cell purification is about 4% of the number of starting cells before transfection. From the 20%–40% of expected transfected cells, we purify 1/5 by this procedure.

### Efficacy of the antitumor vaccine with transfected and selected cells

According to our previous results, total survival of vaccinated animals was obtained with 200.000 non-selected GM-CSF transfected cells. Now, having purified only the B7.2 and GM-CSF producing cells, we vaccinated with a cell dose representing only the expected truly transfected cells, i.e. 40.000 cells (the equivalent of 20% of 200.000), per mouse, per dose. Moreover, we also vaccinated with the double number of cells (80.000) and, since this cell number was still quite reduced, we prepared a mixture of 3LL irradiated cells plus the GMCSF+B7.2 -B16 producing cells to determine wether the procedure could also promote an efficient 3LL antitumor immune response. The treatment groups were: a) Control (100 μl DMEM), to challenge with B16 tumor; b) Control-bis (100 μl DMEM), to challenge with 3LL tumor; c) B16-GMCSF+B7.2/40, employing 40.000 B16 selected cells per dose, in 100 μl DMEM; d) B16-GMCSF+B7.2/80, the same as before but with 80.000 B16 selected cells ; e) B16-GMCSF+B7.2/80 + 3LL, each dose being 80.000 B16 selected cells and 320.000 irradiated 3LL cells, with B16 tumor challenge; f) B16-GMCSF+B7.2/80+3LL, the same as before but with 3LL tumor challenge.

**Figure 3:**
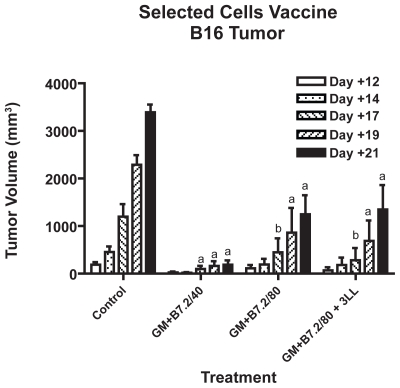
B16 Tumor growth inhibition in the selected cells vaccine Tumor growth inhibition vaccinating with the groups B16-GM+B7.2/40, B16-GM+B7.2/80, B16-GM+B/.2/80 + 3LL and control. Tumor was implanted on day 0 with 10^5^ B16 wild type cells in the left leg and vaccine doses were injected in the right leg on days −21, −7 and +7. “a” represents P < 0.001 and “b” P < 0.01, versus control.

**Figure 4:**
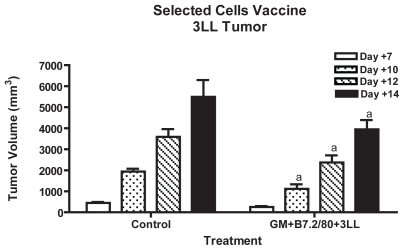
3LL Tumor growth inhibition in the selected cells vaccine Employing the same procedure as in Figure 3, the preventive treatment B16-GM+B7.2/80+3LL (80.000 B16 selected cells + 320.000 3LL irradiated cells) was evaluated versus control, challenging the mice on day 0 with 10^5^ 3LL wild type cells. “a” represents P < 0.001 versus control.

The results of tumor growth inhibition are shown in Figure 3, for the groups with B16 tumor and in Figure 4, for the groups with 3LL tumor.

The three treatment groups of mice bearing B16 tumor were statistically different from the control, with differences equal or bigger than P = 0.01 from day +17. The best B16 tumor inhibition was achieved by the B16-GM+B7.2/40 group, with 90% tumor growth inhibition, versus the control group, on day +21. It is remarkable that no significant differences in tumor growth inhibition were observed between B16-GM+B7.2/80 and B16-GM+B7.2/80 + 3LL groups, suggesting that adding a different type of tumor cell to a bystander cytokine-producing line, does not impair the results.

**Figure 5:**
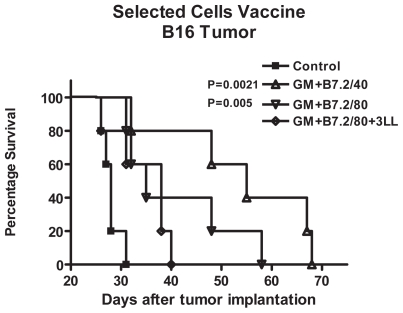
Survival in the selected cells vaccine against B16 tumor With the death dates of the animals, survival curves were constructed with Kaplan-Meier method in the groups with B16 tumor: Control, B16-GM+B7.2/40, B16-GM+B7.2/80 and B16-GM+B7.2/80+3LL. Logrank statistical test was performed and Pvalues are included were significant difference was achieved regarding Control group.

These observations also correlate with the survival curves, in Figure 5, where B16-GM+B7.2/40 achieved the best survival results, doubling the survival period achieved by the control group, with a significant difference of P = 0.0021 with the logrank test. Despite this, no total survival was obtained in any of the mice.

Interestingly, although the same tumor growth inhibition was obtained in both B16-GM+B7.2/80 and B16-GM+B7.2/80+3LL groups, their survival curves show a differentiation of nearly 20 days delayed end-point in the group where no cell mixture had been performed. B16-GM+B7.2/80 achieved a significant difference of P = 0.005 regarding Control group while B16-GM+B7.2/80+3LL showed no statistical difference.

B16-GM+B7.2/40 and B16-GM+B7.2/80 do not show any statistical difference between them.

In the case of mice bearing 3LL tumor, the total time of survival was very short, 20 days maximum, since 3LL is a highly aggressive tumor. Although the treated group achieved significant tumor growth inhibition with P < 0.001 from day +10, versus its control, the volume reduction was about 20% maximum, which was not enough to diferentiate the survival curves of the two groups (data not shown).

### Specific anti-TMP IgG production

**Figure 6:**
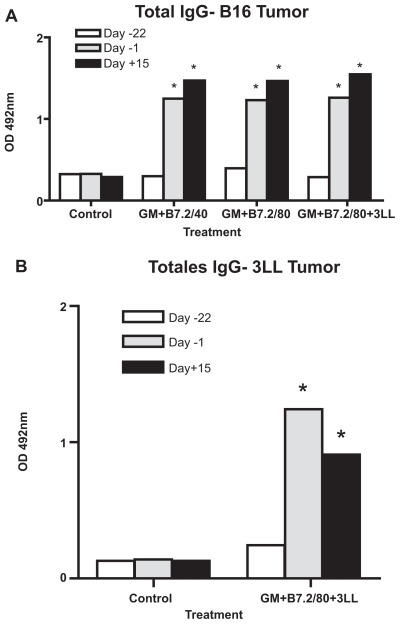
Total anti-TMP IgG Blood was extracted from the tail vein on days −22, −1 and +15 regarding tumor implantation on day 0. ELISA assays were performed in the plasma obtained. Figure 6-A shows the results in the groups challenged with B16 tumor and Figure 6-B, those for 3LL tumor. The samples were analyzed by duplicate with representation of the mean OD and SD, though SD cannot be appreciated since it was very small. Two-way ANOVA was performed, *P < 0.001.

The findings from the anti-TMP ELISA studies in measuring specific total IgG production are summarized in Figure 6-A, for results in the B16 tumor groups and Figure 6-B, for 3LL tumor groups. In the total specific IgG studies, we always found higher productions in the treated groups compared with the controls. In the B16 tumor groups, there were no remarkable differences and all of them peaked on day +15. However, the group treated against 3LL showed its maximum on day −1. In all cases, the SD was less than 5% of the OD mean value, and therefore the SD is not appreciable in the graphs. After performing the two-way ANOVA test, all the treatments were statistically different from the Control group at days −1 and +15, with P < 0.001.

**Figure 7:**
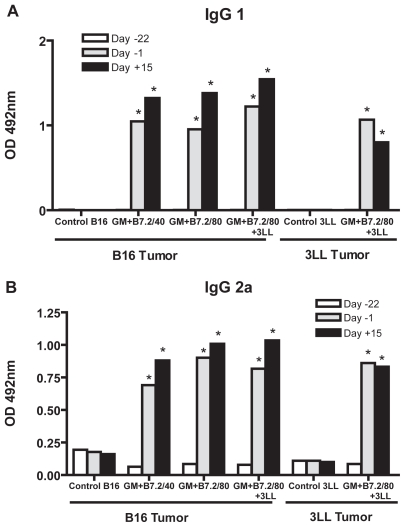
Anti-TMP IgG subtypes In the same way as in Fig. 6, the plasma samples were tested with ELISA for IgG subtypes, IgG1 in Figure 7-A and IgG2a in Figure 7-B. Two-way ANOVA was performed, *P < 0.001.

The same results were observed in the IgG subtypes IgG1 (Fig. 7-A) and IgG2a (Fig. 7-B) analysis.

## Discussion

Engineered cells secreting cytokines have demonstrated to be a very interesting approach in cancer vaccines (Dunussi-Joannopoulos et al. 1998; Jaffee et al. 1998; Shi et al. 1999; Kim et al. 2000; Mastrangelo and Lattime, 2002; Borrello and Pardoll, 2002; Serafini et al. 2004), as we have also seen (Moret-Tatay et al. 2003; Herrero et al. 2006; Moret-Tatay et al. 2006; Herrero et al. 2008), though we are aware of the great difficulty of working with this approach in actual clinical practice. In fact, tissue or cell samples from the patients must be kept in culture, amplified to a larger number, transfected and then returned to the same patient. In this context, guaranteeing strict handling conditions for clinical use, an acceptable number of cells, as well as cytokine production, is not an easy task. This is why we decided to try to reduce the cell number required to start a proper immune response against the tumor, thus avoiding very complex cell work in future patients.

In our hands, the best results of preventive antitumor vaccines were obtained in a murine melanoma model with a vaccine dose of 2 × 10^5^ transfected cells. The vaccination protocol was exactly the same as described in this manuscript. With that vaccine we achieved 100% and 80% survival levels employing genetically modified cells to produce mGM-CSF and mGMCSF+mB7.2, respectively. In those experiments we confirmed two points, supported also by the findings of other groups: 1) The idea that larger levels of GM-CSF production are not necessarily better (Borrello and Pardoll, 2002; Rodríguez-Lecompte et al. 2004; Serafini et al. 2004; Herrero et al. 2006) but are sometimes worse; and 2) There is some kind of synergistic effect between GM-CSF and B7.2. Thus, we knew that we did not really need an enormous quantity of GM-CSF, and that B7.2 also offered some advantages in several tumor vaccine models. Finally, we knew that only 20%–40% of our cells were really functional, because this was the true transfection efficacy. Thus, the question is: Why not try a vaccine with only the truly transfected cells? Many efforts have been made in the course of gene therapy history to achieve transfection of the totality of the cells. However, to date this has been nearly impossible, particularly when working with non-viral vectors, although the different methods have been greatly improved (i.e Amaxa’s Nucleofector). Our approach is not to enhance the transfection process, but to isolate and purify the transfected cells that we obtain. We employed the Automacs (Miltenyi Biotec, Spain) to select our cells producing both GM-CSF and B7.2, using the magnetic streptavidin microbeads protocol.

Following this protocol, 72 h after cell transfection, we purified 4% viable cells from the total cells that we started with. We think that this is quite a low yield, since the transfected cells are supposed to represent 20%–40% of the starting cells (Herrero et al. 2008). These results could be due to several reasons. On one hand, the whole purification process is time consuming (several hours), during which the cells probably suffer from not being in their appropriate culture conditions. This results in a dramatic drop in viability. Therefore, optimized protocol conditions for reducing the length of the process or the kind of selection protocol in the device would contribute to improve the cell selection yield. Another reason to take in consideration is that probably, our B7.2 cassette, which provides long term expression, is not really strong since the fluorescence values in Figure 2 are not really high. Probably, using a more potent promoter could boost B7.2 expression and then easier cell recognition and separation in the Automacs would be achieved.

We studied two different purified cell doses in a preventive vaccine model, 40.000 selected cells (20% of 2 × 10^5^) and 80.000 selected cells (40% of 2 × 10^5^), in an attempt to reproduce the successful model that we achieved with preventive non-selected genetically modified cells but this time employing only the number of truly transfected cells that we expected to have in the previous assays. With this new approach, we reached a maximum of 90% tumor growth inhibition in the group treated with B16-GM+B7.2/40 against B16 melanoma tumor. The groups treated with B16-GM+B7.2/80 and B16-GM+B7.2/80 + 3LL, contributing double dose of cell antigen, GM-CSF and B7.2, surprisingly only reached about 60% tumor growth inhibition. As other authors have pointed out (Borrello and Pardoll, 2002; Serafini et al. 2004), this suggests that here we probably have an excess of GM-CSF, which proves disadvantageous in our system. A vaccine assay exploring dose-dependent effects of our selected cells would help us verify this point. Phenomena of immune suppression have been described in the last few years to explain results of this kind and the apparent paradoxical effect of certain cytokines as GM-CSF, that can act as immune activators or, contrarily, as suppressors of the immune response. This is most probably due to immune cells such as regulatory T cells (De Visser et al. 2006; Zou, 2006) or the recently described Myeloid Derived Suppressor Cells (Serafini et al. 2004; Horna et al. 2006; Serafini et al. 2006a, b). The survival results correlate with those of tumor growth inhibition: although no total survival was achieved, the best results were obtained with B16-GM+B7.2/40, doubling the survival period of the control group. The other two groups challenged with B16 tumor had different survival results despite similar tumor growth inhibitions. This could also be due to the suppressor phenomena that have been mentioned above, where the “extra” irradiated cells could be generating an extra immune response that impairs the action against B16 tumor.

We also wanted to take advantage of working with a reduced number of cells, to test a new possibility, that could be very interesting in the clinical practice: mixing our transfected and purified B16 cells, in the role of cytokine producers and non specific immune stimulants, with other different cells, 3LL in this case, in the role of specific immunizing antigen, for combating a 3LL tumor. This first approach could also be intended for other bystander cytokine producing non-tumor cells, as for instance fibroblasts, which would be easier to remove and reintroduce to a clinical patient. We achieved a significant tumor reduction (approximately 25%) in the treated group, though this was not good enough to elicit any kind of survival. 3LL tumor is a very aggressive and a rapidly progressing malignancy; as a result, by better adjusting the doses and times, we probably would obtain better results.

Regarding specific IgG productions, we always found higher levels in treated groups than in controls, though interestingly, in the groups challenged with B16 tumor, which worked better, we recorded the highest values on day +15, while in groups challenged with 3LL tumor, which worked worse, the highest values appeared on day −1. Again, these results suggest a different kind of immunoglobulin switch and therefore, a different sequence of immune responses, resulting in very different rates of success (Herrero et al. 2006).

Although the overall results were not as good as those with the non-selected cells vaccine, no conclusions can be drawn before a dose-dependent study is performed with these selected cells. It remains to be elucidated how synergistic effects between antigens, GM-CSF and costimulatory B7.2 molecules cooperate in the generation of efficient antitumor response or toxicity. We cannot exclude a lack of net antigen contribution due to the reduction in cell number or, contrarily, an excess in GM-CSF production which could result in a loss of vaccine efficacy. These experiments will focus our future attention, and will contribute to clarify the exact need for each component in a cell vaccine that could become suitable for use in clinical practice.
